# Utilization of *N*-Bromosuccinimide as a Brominating Agent for the Determination of Sumatriptan Succinate in Bulk Drug and Tablets

**DOI:** 10.1155/2013/934357

**Published:** 2013-07-09

**Authors:** Kudige N. Prashanth, Kanakapura Basavaiah, Madihalli S. Raghu

**Affiliations:** Department of Chemistry, University of Mysore, Manasagangotri, Mysore, Karnataka 570006, India

## Abstract

One titrimetric and two spectrophotometric methods which are simple, sensitive, and economic are described for the determination of sumatriptan succinate (STS) in bulk drug and in tablet dosage form using *N*-bromosuccinimide (NBS) as a brominating agent. In titrimetry, aqueous solution of STS is treated with a measured excess of NBS in acetic acid medium, and after the bromination of STS is judged to be complete, the unreacted NBS is determined iodometrically (method A). Spectrophotometric methods entail addition of a known excess of NBS in acid medium followed by the determination of residual NBS by its reaction with excess iodide, and the liberated iodine (I_3_^−^) is either measured at 370 nm (method B) or liberated iodine is reacted with starch followed by the measurement of the blue colored starch-iodine complex at 570 nm (method C). Titrimetric method is applicable over range 1.0–10.0 mg STS (method A), and the reaction stoichiometry is found to be 1 : 3 (STS : NBS). The spectrophotometric methods obey Beer's law for concentration range 0.6–15.0 **μ**g mL^−1^ (method B) and 0.2–4.0 **μ**g mL^−1^ (method C). The calculated apparent molar absorptivity values were found to be 2.10 × 10^4^ and 7.44 × 10^4^ L mol^−1^ cm^−1^, for method B and method C, respectively.

## 1. Introduction

**Figure 1:**
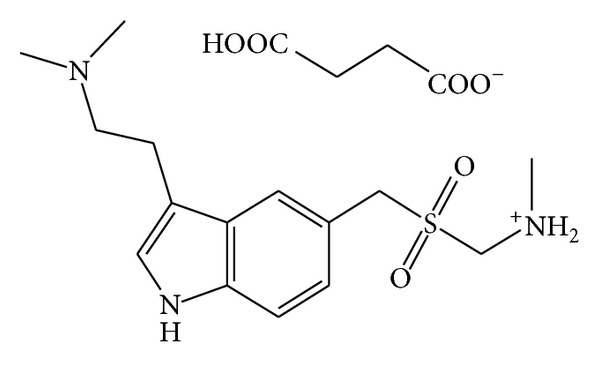
Structure of STS.

Triptans are a group of tryptamine-based drugs used in the acute treatment of migraine headaches. Sumatriptan succinate (Figure 1) is one among them and is structurally related to the neurotransmitter serotonin. Sumatriptan succinate (STS) is a 5-hydroxytryptamine (5-HT) receptor subtype (a member of the 5-HT 1D family) having only a week affinity for 5-HT_1A_, 5-HT_5A_, and 5-HT_7_ receptors and chemically designated as [3-[2-(dimethylamino)ethyl]-1H-indol-5-yl]-N-methylmethanesulphonamide hydrogen butanedioate [1]. STS acts by selectively binding to serotonin type-1D receptors (serotonin agonist) and rapidly terminates a migraine attack while eliminating associated symptoms such as nausea, vomiting, and light and sound sensitivity [2].

 STS has official monographs in BP [1], EP [3], and USP [4] which describe liquid chromatographic methods for the assay of STS. From the literature survey, it is found that high performance liquid chromatography (HPLC) has been used for the assay of STS in human plasma [5, 6], human serum [7], rabbit plasma [8], and human plasma and urine [9] whereas liquid chromatography-mass spectrophotometry is used (LC-MS/MS) in body fluids [10] and human plasma [11]. Several methods have been reported for the determination of STS in pharmaceuticals and include UV-spectrophotometry [12–16], HPLC [17–20], ultra performance liquid chromatography (UPLC) [21], high performance thin layer chromatography (HPTLC) [16, 22], capillary electrophoresis [23], micellar electrokinetic chromatography [24], and voltammetry [25–27].

 Besides, STS in pharmaceuticals is reported to have been determined by visible spectrophotometry employing different reaction schemes. Two methods were reported by Kalyanaramu and Raghubabu [28]; first method was based on oxidative coupling of STS with brucine in presence of sodium metaperiodate, and the second method was based on the formation of internal salt between drug and aconitic anhydride, dehydration product of citric acid with acetic anhydride. The same authors reported a method based on the formation of inner molecular complex between drug and sodium nitroprusside in acetaldehyde [29]. A green colored ternary complex formed by the drug with cobalt-thiocyanate was extracted into benzene and measured at 630 nm and served as the basis of its assay [30]. Tropaeolin 000 is reported to form chloroform extractable orange-colored ion-pair with STS having an absorption maximum at 483 nm, and this was used for the sensitive assay of the drug by Kalyanaramu and Raghubabu [31]. 

Kalyanaramu et al. [32] reported two methods based on charge transfer reaction. The first method is based on the formation of charge transfer complex between the drug and chloranil, while the second method is based on the interaction of N-alkyl vinyl amine formed from the condensation of the free secondary amine group in the drug and acetaldehyde with p-chloranil to give vinyl amino substituted quinone. Fathima et al. [33] reported a method based on C-T complexation reaction of drug with p-chloranilic acid. The reaction between STS and sodium salt of 1,2-naphthaquinone-4-sulphonic acid (Folin reagent) yielded a brown colored chromogen [34] forming the basis for the assay of the drug. Based on a well-known redox reaction and employing Folin-Ciocalteu's reagent [35], the drug in pharmaceutical dosage forms was determined by Tipre and Vivia. Satyanarayana and Rao [36] have described two methods using the reaction between drug and *in situ* bromine and the unreacted bromine measured either by methyl orange or indigo carmine. 

 No titrimetric method was found in the literature for the quantification of STS in pharmaceuticals. The reported visible spectrophotometric methods suffer from one or more disadvantages such as rigid pH control, heating and/or extraction step, use of multistep reaction/s, longer contact time, less stable colored species, and narrow linear dynamic range as indicated in Table 1.

The present paper describes one titrimetric and two visible spectrophotometric methods based on the bromination reaction by NBS. Simplicity, sensitivity, wide linear ranges, mild experimental conditions, and above all cost-effectiveness characterize the proposed methods. Optimum conditions were established and all the methods were validated according to ICH guidelines. The validated methods when applied to the determination of STS in tablets yielded results which were in good agreement with the label claim.

## 2. Experiment

### 2.1. Apparatus

A Systronics model 106 digital spectrophotometer with 1 cm path length matched quartz cells was used to record the absorbance values.

### 2.2. Reagents and Standards

All chemicals used were of analytical reagent grade. Distilled water was used throughout the investigation.

*N-Bromosuccinimide (0.01 M, 200 *μ*g mL* ^−1^ *and 60 *μ*g mL* ^−1^ *).* An approximately 0.01 M solution was prepared by dissolving about 1.8 g of NBS (SRL Research Chemicals, Mumbai, India) in water with the aid of heat and diluted to one liter water. The solution was standardized iodometrically [37] and kept in an amber colored bottle and stored in a refrigerator and used in titrimetry. It was diluted appropriately to get working concentrations of 200 and 60 *μ*g mL^−1^ NBS for use in spectrophotometric method B and method C, respectively. 

*Potassium Iodide.* A 5% potassium iodide (Merck, Mumbai, India) solution was prepared by dissolving 5 g potassium iodide with water in a 100 mL calibrated flask. This solution was prepared afresh daily. A 2% solution was prepared separately for spectrophotometric work.

*Starch Solution.* One gram of starch (LOBA Chemie Ltd., Mumbai, India) was made into paste with water and slowly poured with constant stirring into 100 mL boiling water, boiled for 5 min, cooled, and used. This solution was prepared freshly every day.

*Hydrochloric Acid.* Concentrated hydrochloric acid (Merck, Mumbai, India, Sp. gr. 1.18) was diluted appropriately with water to get 2 M HCl for use in all the methods.

*Sodium Acetate.* A 3 M aqueous solution of sodium acetate was prepared by dissolving suitable quantity of sodium acetate trihydrate crystals (Merck, Mumbai, India) in water for use in spectrophotometric method B.

*Standard Solution of STS.* Pharmaceutical grade sumatriptan succinate sample (purity 99.5%) was received from MSN laboratories, Hyderabad, India, as gift sample and was used as received. A stock standard solution equivalent to 1.0 mg mL^−1^ of STS was prepared by dissolving accurately weighed 250 mg of pure drug in water and diluted to mark in a 250 mL calibrated flask with the same solvent. The solution (1 mg mL^−1^ STS) was used in titrimetric work and diluted appropriately with water to get the working concentrations of 30 and 10 *μ*g mL^−1^ STS for use in spectrophotometric method B and method C, respectively. 

Two brands of tablets, namely, Suminat-25 and Suminat-50 (Sun Pharmaceuticals Ltd., Sikkim, India), both were purchased from local commercial sources.

### 2.3. General Procedures

#### 2.3.1. Titrimetry (Method A)

A 10.0 mL aliquot of pure drug solution containing 1.0–10.0 mg of STS was measured accurately and transferred into a 100 mL Erlenmeyer flask. The solution was acidified by adding 5 mL of 2.0 M HCl. Then 10 mL of 0.01 M NBS was added by means of a pipette and the flask was let stand for 15 min at room temperature. Lastly, 5 mL of 5% KI was added, and after 5 min the liberated iodine was titrated against 0.02 M sodium thiosulphate using 1 mL of 1% starch as an indicator. A blank experiment was simultaneously performed.

The amount of STS was computed from the following formula:

(1) Amount  (mg)=(B−A)×Mr×Sn,

where *B* is mL of titrant in the absence of sample, *A* is mL of titrant in the presence of the sample, *M*_*r*_ is relative molecular mass of drug, *S* is strength of NBS, and *n* is number of moles of titrant reacting with per mole of STS.

#### 2.3.2. Spectrophotometric Method B (Based on the Measurement of Tri-Iodide Ion)

Varying aliquots (0.2–5.0 mL) of standard STS solution (30 *μ*g mL^−1^) were accurately transferred into a series of 10 mL calibrated flasks and the total volume was adjusted to 5.0 mL with water. One mL of 2 M HCl was added to each flask followed by the addition of 1 mL NBS solution (200 *μ*g mL^−1^ in NBS). The content was mixed well and let stand for 15 min with occasional shaking. Then, 1.0 mL of 3 M sodium acetate solution was added to each flask followed by 1 mL of 2% potassium iodide. The volume was brought to the mark with water and the absorbance of the resulting tri-iodide ion was measured at 370 nm after 5 min against water blank.

#### 2.3.3. Spectrophotometric Method C (Based on the Measurement of Starch-Iodine Complex)

Into a series of 10 mL calibrated flasks, different aliquots (0.2–4.0 mL) of standard STS (10 *μ*g mL^−1^) solution were transferred using a microburette. The total volume in each flask was brought to 4 mL by adding required quantity of water. The solution was acidified by adding 1 mL of 2 M HCl, and 1 mL of NBS (60 *μ*g mL^−1^ in NBS) solution was then added to each flask. The flasks were kept aside for 15 min with periodic shaking; 1 mL of 2% potassium iodide was added and the content was mixed well. After 5 min, 1 mL of 1% starch solution was added to each flask and the volume was made up to the mark with water and mixed well. The absorbance of the resulting blue chromogen was measured at 570 nm against water blank after 5 min. 

A standard graph was prepared by plotting absorbance against concentration and the unknown concentration was read from the graph or computed from the regression equation derived using Beer's law data.

#### 2.3.4. Assay Procedure for Tablets

Twenty tablets each containing 25 or 50 mg of STS were weighed accurately and ground into a fine powder. To prepare a standard stock solution of tablet, an amount of the powder equivalent to 100 mg of STS was accurately weighed into a 100 mL volumetric flask, and 60 mL water was added and content shaken thoroughly for about 20 min. The volume was diluted to the mark with water, mixed well, and filtered using Whatman no. 42 filter paper. First 10 mL portion of the filtrate was rejected and a convenient aliquot of filtrate (containing 1 mg mL^−1^ STS) was taken for assay by titrimetric procedure. Further, the standard stock solution of tablet was diluted stepwise to achieve 30 and 10 *μ*g mL^−1^ of STS for use in spectrophotometric method B and method C, respectively. A suitable aliquot was then subjected to analysis following the procedures described earlier.

#### 2.3.5. Procedure for the Analysis of Placebo Blank and Synthetic Mixture

A placebo blank containing starch (10 mg), acacia (15 mg), hydroxyl cellulose (20 mg), sodium citrate (30 mg), lactose (10 mg), talc (60 mg), acacia (30 mg), magnesium stearate (25 mg), and sodium alginate (30 mg) was prepared, and 20 mg of the placebo blank was extracted with water and the solution was made as described under “assay procedure for tablets” and then subjected to analysis. 

A synthetic mixture was prepared by adding 50 mg of STS to about 25 mg of the placebo blank prepared earlier, homogenized, and the solution was prepared as done under “assay procedure for tablets.” The filtrate was collected in a 50 mL flask. The synthetic mixture solution was analysed by titrimetry and then appropriately diluted with water to get 30.0 and 10.0 *μ*g mL^−1^ STS solutions, and appropriate aliquots were subjected to analysis by method B and method C, separately.

## 3. Results and Discussion

### 3.1. Chemistry

**Scheme 1:**
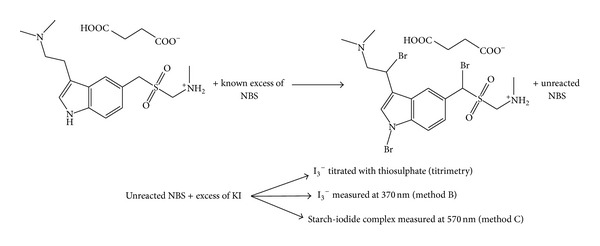
Proposed reaction pathway for the bromination of STS by NBS.

NBS is widely used as an oxidizing or brominating reagent for the determination of many pharmaceutically important compounds [38–41]. It is also a specific reagent for the bromination of organic compounds at allylic position [42]. As NBS is a mild oxidizing agent, the only possible reaction between STS and NBS would be bromination at the two allylic positions and a secondary amine present in pyrrole ring present in STS. This was supported by a reaction stoichiometry of 1 : 3 between STS and NBS. The possible reaction scheme is presented in Scheme 1. The proposed methods are indirect and involve the determination of unreacted NBS after allowing the reaction between STS and a measured amount of NBS go to completion. The amount of iodine liberated, by the reaction of unreacted NBS with potassium iodide, was either measured directly at 370 nm or reacted with starch, and resulting blue colored chromogen of starch-iodine complex was measured at 570 nm whereas iodometric back titration procedure was used in titrimetry.

### 3.2. Optimization of Reaction Conditions

#### 3.2.1. Titrimetry

Direct titration of STS with NBS was not feasible due to slow reaction. In the indirect procedure, a known excess of NBS was allowed to react with STS in acid medium and the unreacted NBS was subsequently determined iodometrically. At optimum acid concentration (5 mL of 2 M HCl in a total volume of 25 mL), the reaction goes to completion within 15 min. At lower acid concentration (less than 3 mL of 5 M HCl) the reaction stoichiometry was slightly less and at higher acid concentration (up to 8 mL of 5 M HCl) regular stoichiometry was observed. Under the optimized reaction condition, there was found to be a definite reaction stoichiometry of 1 : 3 between STS and NBS within the range of  1.0–10.0 mg STS. Contact time of 5 minute, was sufficient for the complete liberation of iodine from the unreacted NBS. 

#### 3.2.2. Spectrophotometry

**Figure 2:**
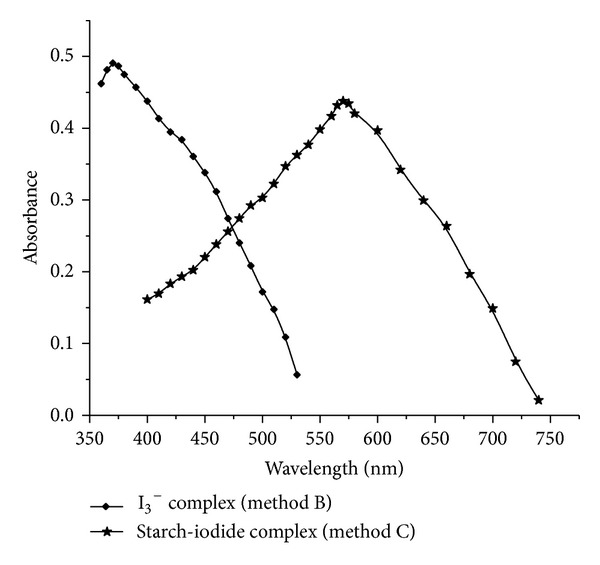
Absorption spectra: 6.0 *μ*g mL^−1^ STS, tri-iodate (method B); 2.0 *μ*g mL^−1^ STS, starch-iodide complex (method C).

In spectrophotometric methods, the amount of iodine liberated, by the reaction of unreacted bromine with potassium iodide, was either measured directly at 370 in method B or reacted with starch, and resulting blue colored chromogen of starch-iodide complex is measured at 570 nm for method C (Figure 2). 

*Optimization of NBS. *To fix the optimum concentration of NBS, different concentrations of NBS were treated with a fixed concentration (1 mL 2% KI) of potassium iodide in method B and potassium iodide and starch (1 mL 1% starch) in method C in HCl medium and the absorbance was measured at 370 nm and 570 nm, respectively. A constant and maximum absorbance resulted in 20 *μ*g mL^−1^ NBS in method B and 6 *μ*g mL^−1^ NBS in method C. Hence, different concentrations of STS were treated with 1 mL of 200 *μ*g mL^−1^ NBS (method B) and 60 *μ*g mL^−1^ NBS (method C) in HCl medium before determining the residual NBS as illustrated in the reaction Scheme 1. This facilitated the optimization of the linear dynamic ranges over which procedure could be applied for the assay of STS. 

**Figure 3:**
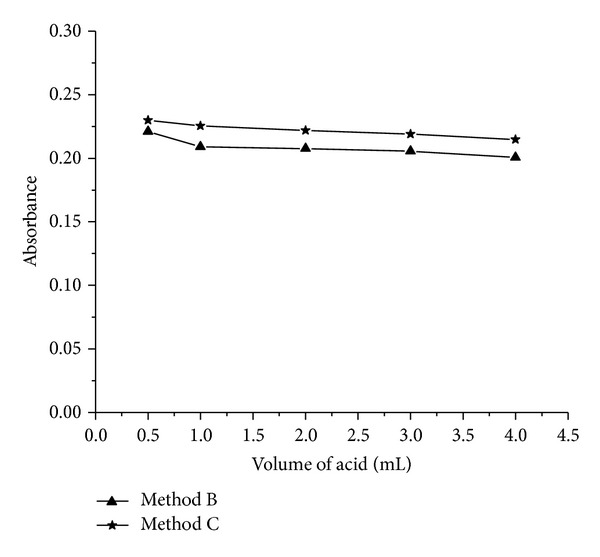
Effect acid concentration on 12 *μ*g mL^−1^ STS (method B) and 3 *μ*g mL^−1^ STS (method C).

*Effect of Acid Concentration. *The reaction between STS and NBS was performed in different acid media. Better results were obtained in hydrochloric acid medium. The effect of acid concentration on the reaction between STS and NBS was studied by varying the concentration of HCl keeping the concentrations of NBS and drug fixed. The reaction was found to be rapid yielding a constant absorbance with maximum sensitivity and stability when the HCl concentration was maintained in the range of 0.18–0.75 M (0.5 to 3.0 mL 2 M HCl). Therefore, 1 mL of 2 M HCl in a total volume of 6 mL (0.33 M) was used in both methods (Figure 3).

*Reaction Time and Color Stability. *The effect of time on the reaction between STS and NBS in the presence of HCl was studied by keeping all other reaction conditions unchanged. The absorbance of the colored species was measured after different reaction times (5.0–30.0 min) and the results showed that the reaction was complete within 15 min in both method B and C. The absorbance of yellow tri-iodide ion in method B and starch-iodide complex chromogen in method C remained stable for at least 1 hr. 

*Role of Sodium Acetate. *The liberation of iodine in method B did not stop even after 30 min under the specified acidic conditions, but on adding sodium acetate the reaction ceased immediately. The amount of sodium acetate required was optimized as 1 mL of 3 M sodium acetate in a total volume of 10 mL. In method C, the reaction was ceased by optimum amount of starch (1 mL of 1% starch).

### 3.3. Method Validation

The proposed methods have been validated for linearity, sensitivity, selectivity, precision, accuracy, and recovery according ICH guidelines [43]. 

#### 3.3.1. Linearity, Detection, and Quantification Limits

The reaction stoichiometry for the titrimetric procedure was 1 : 3 owing to the presence of two allylic groups and a secondary aromatic amine in STS and the range of quantification was 1.0–10.0 mg STS. A linear correlation was found between absorbance and concentration of STS in the spectrophotometric methods. Regression analysis of Beer's law data using the method of least squares was made to evaluate the slope (*b*), intercept (*a*), and the correlation coefficient (*r*) and the calculated values are given in Table 2. The graph showed negligible intercept and is described by the regression equation *y* = *a* + *bx*, where *y* is the measured absorbance and *x* concentration in *μ*g mL^−1^. The limits of detection (LOD) and quantification (LOQ), sensitivity parameters such as molar absorptivity, and Sandell sensitivity are also contained in Table 2. 

#### 3.3.2. Accuracy and Precision

The accuracy of an analytical method expresses the closeness between the reference value and the found value. Accuracy was evaluated as percentage relative error between the measured concentrations and taken concentrations for STS (Bias%). The results obtained are compiled in Table 3 and show that the accuracy is good for the methods.

 The precision of the method was calculated in terms of intermediate precision (intra-day and inter-day). Three different concentrations of STS were analyzed in seven replicates during the same day (intra-day precision) and five consecutive days (inter-day precision). The RSD (%) values of intra-day and inter-day studies showed that the precision was good (Table 3).

#### 3.3.3. Robustness and Ruggedness

To evaluate the robustness of the methods, volume of HCl was slightly altered (5 ± 1 mL) with reference to optimum values in titrimetry. However, in spectrophotometry, the reaction time (after adding NBS, time varied was 15 ± 1 min in both methods B and C) and volume of 2 M HCl were slightly altered (1 ± 0.1 mL) in both B and C methods. To check the ruggedness, analysis was performed using four different burettes in method A and three different cuvettes in methods B and C. The robustness and the ruggedness were checked at three different drug levels (4, 6, 8 mg in method A; 6, 9, 12 *μ*g mL^−1^ in method B; and 1, 2, 3 *μ*g mL^−1^ in method C). The intermediate precision, expressed as percent RSD, which is a measure of robustness and ruggedness, was within the acceptable limits (0.89–2.27%) as shown in Table 4.

#### 3.3.4. Selectivity

The proposed methods were tested for selectivity by placebo blank and synthetic mixture analyses. A convenient aliquot of the placebo blank solution, prepared as described earlier, was subjected to analysis by titrimetry and spectrophotometry according to the general procedures. In all the cases, there was no interference by the inactive ingredients present in the placebo mixture. 

A separate experiment was performed with the synthetic mixture. The analysis of synthetic mixture solution prepared earlier yielded percent recoveries which ranged from 98.77 to 102.6 with standard deviation of 0.89–1.74 in all the cases. The results of this study indicate that the inactive ingredients present in the synthetic mixture did not interfere in the assay. These results further demonstrate the accuracy, as well as the precision, of the proposed methods. 

#### 3.3.5. Application to Tablets Analysis

The proposed methods were applied to determine STS in same brand of tablets with two different doses. The results were statically compared with those obtained by the published reference method [12] for accuracy and precision by applying the Student's *t*-test and variance ratio *F*-test. The published reference method describes UV-spectrophotometric method for detection of STS in tablet formulation at 220 nm. Statistical analysis of the results using Student's *t*-test for accuracy and *F*-test for precision revealed no significant difference between the proposed methods and the reference method at the 95% confidence level with respect to accuracy and precision (Table 5).

#### 3.3.6. Recovery Studies

The accuracy and validity of the proposed methods were further ascertained by performing recovery studies. Preanalysed tablet powder was spiked with pure STS at three concentration levels (50%, 100%, and 150% of that in tablet powder) and the total was found by the proposed methods. The added STS recovery percent values ranged between 98.20% and 103.0% with a standard deviation of 0.05–1.56. The results of this study given in Table 6 indicated that the recovery was good, and that the coformulated substances did not interfere in the determination.

## 4. Conclusions

Three simple, sensitive, and selective methods are described for the determination of STS in tablets employing NBS as the brominating reagent. Titrimetry is applicable over 1.0–10.0 mg range, and it is the first titrimetric method has ever been reported for the assay of STS in pharmaceuticals. The proposed spectrophotometric methods are characterized by high sensitivity and stability of the measured colored species. The proposed spectrophotometric methods are more sensitive than most of the reported methods in terms of both linear range and molar absorptivity. The proposed methods are simple, rapid, economic, and not involved in strict pH control and use of organic solvents, whereas most of the reported methods require rigid pH control, extraction step, and multistep reactions. Besides, the methods possess adequate accuracy and precision, and no interference from the excipients and coating materials was found. Hence, the methods can be employed for routine analysis in quality control laboratories. 

## Figures and Tables

**Table 1:** Comparison of the proposed and the existing visible spectrophotometric methods.

Sl. no.	Reagent/s	Methodology	*λ*_max⁡_, nm	Beer's law range *µ*g mL^−1^ (*ε* in L mol^−1^ cm^−1^)	Remarks	Reference
1	(a) Brucine-sodium metaperiodate	Oxidative coupling product was measured	520	4.0–20.0 NA	Less sensitive, multistep reaction	[28]
	(b) Citric acid-acetic anhydride		580	8.0–24.0 NA		
2	Sodium nitroprusside acetaldehyde	Inner molecular complex formed was measured	552	4.0–20.0 (1.10 × 10^4^)	Less sensitive, requires rigid pH control	[29]
3	Cobalt thiocyanate	Extracted ternary complex formed by reaction with drug was measured	629.4	16.0–48.0 (3.97 × 10^3^)	Less sensitive, involves extraction step	[30]
4	Tropaeolin-OOO	Extracted ion-pair complex was measured	482.5	2.0–10.0 (2.08 × 10^4^)	Requires rigid pH control; involves liquid-liquid extraction; use of organic solvents	[31]
5	(a) Chloranil in 1,4-dioxane	CT-complex measured	548	5.0–25.0 (1.00 × 10^4^)	Less sensitive, involves heating step, time consuming	[32]
	(b) Chloranil and acetaldehyde		660	20.0–60.0 (3.19 × 10^4^)		
6	p-Chloranilic acid	CT-complex measured	520	9.28 × 10^2^ 20–184	Less sensitive; use of organic solvents	[33]
7	Folin reagent	Chromogen formed by reaction with drug was measured	455.6	16.0–48.0 (3.85 × 10^3^)	Less sensitive, strict pH control, time consuming	[34]
8	Folin-Ciocaltaeu reagent	Reduced FC-reagent was measured	760	2.0–6.0 NA	Narrow linear range, less sensitive	[35]
9	Bromate-bromide-					
	(a) Methyl orange	Unreacted bromine was measured	508	0.2–1.6 (1.90 × 10^5^)	Narrow linear range, multistep reaction, time consuming	[36]
	(b) Indigo carmine		610	2.0–12.0 (2.71 × 10^4^)		
10	NBS				No rigorous control of experimental conditions, no heating or extraction step, no use of organic solvents or toxic chemicals, and sensitive with wide linear dynamic range	Proposed methods
	(a) Potassium Iodide	Tri-iodide ion measured Starch-iodine complex measured	370	0.6–15.0 (*ε* = 2.10 × 10^4^)		
	(b) Potassium Iodide-starch		570	0.2–4.0 (*ε* = 7.44 × 10^4^)		

NBS: *N*-bromosuccinimide. NA: not available.

**Table 2:** Regression and analytical parameters.

Parameter	Method B	Method C
*λ*_max⁡_, nm	370	570
Beer's law limits, *µ*g mL^−1^	0.0–15.0	0.0–4.0
Molar absorptivity (*ɛ*), L mol^−1^ cm^−1^	2.10 × 10^4^	7.44 × 10^4^
Sandell sensitivity^a^, *µ*g cm^−2^	0.0197	0.0056
Limit of detection (LOD), *µ*g mL^−1^	0.34	0.07
Limit of quantification (LOQ), *µ*g mL^−1^	1.02	0.20
Regression equation, *Y* ^b^		
Intercept (*a*)	0.79	0.80
Slope (*b*)	−0.049	−0.19
Correlation coefficient (*r*)	0.9996	0.9997
Standard deviation of intercept (*S*_*a*_)	0.074	0.078
Standard deviation of slope (*S*_*b*_)	0.009	0.037

^a^Limit of determination as the weight in *μ*g per mL of solution, which corresponds to an absorbance of *A* = 0.001 measured in a cuvette of cross-sectional area 1 cm^2^ and *l* = 1 cm.

^b^ *Y* = *a* + *bX*, where *Y* is the absorbance, *X* is concentration in *μ*g mL^−1^, *a* is intercept, and *b* is slope.

**Table 3:** Evaluation of intra-day and inter-day precision and accuracy.

Method	STS* taken	Intra-day (*n* = 5)			Inter-day (*n* = 5)		
		STS found^a^	%RSD^b^	%RE^c^	STS found^a^	%RSD^b^	%RE^c^
A (Titrimetry)	4.00	3.98	0.90	0.55	3.95	1.39	1.21
	6.00	5.99	0.92	0.23	6.04	1.09	0.65
	8.00	8.05	0.73	0.59	8.06	1.06	0.76

B (Spectrophotometry)	6.00	6.06	0.97	0.96	6.08	1.83	1.42
	9.00	9.10	1.04	1.06	9.12	1.57	1.32
	12.0	11.9	1.02	0.94	11.9	1.40	1.12

C (Spectrophotometry)	1.00	1.02	1.44	1.55	1.02	2.20	1.93
	2.00	1.98	0.94	1.14	1.97	1.37	1.40
	3.00	2.97	0.63	0.85	2.97	0.91	1.02

*In method A, STS taken/found are in mg and they are *μ*g mL^−1^ in methods B and C.

^a^Mean value of five determinations.

^b^Relative standard deviation (%).

^c^Relative error (%).

**Table 4:** Robustness and ruggedness.

Method A			Method B				Method C			
STS studied mg	Robustness (RSD, %) Volume of HCl^a^ (*n* = 3)	Ruggedness (RSD, %) Interburettes (*n* = 4)	STS studied *µ*g mL^−1^	Robustness (RSD, %) Conditions altered		Ruggedness (RSD, %)	STS studied *µ*g mL^−1^	Robustness (RSD, %) Conditions altered		Ruggedness (RSD, %)
				Volume of HCl^a^ (*n* = 3)	Reaction time^b^ (*n* = 3)	Intercuvettes (*n* = 3)		Volume of HCl^a^ (*n* = 3)	Reaction time^b^ (*n* = 3)	Intercuvettes (*n* = 3)
4.00	0.77	1.15	6.00	1.70	1.95	2.27	1.00	1.39	1.51	1.93
6.00	1.01	0.89	9.00	2.12	1.72	2.15	2.00	1.86	1.83	2.08
8.00	0.93	1.00	12.0	1.37	1.44	1.97	3.00	1.40	1.28	1.89

^a^In method A, volumes of 2 M HCl varied were 5 ± 1 mL; in both methods B and C, 2 M HCl (1 mL ± 0.1 mL).

^b^In both methods B and C, the reaction time employed was 15 ± 1 min.

**Table 5:** Results of analysis of tablets by the proposed methods.

Tablet brand name	Label claim mg/tablet	Found (percent of label claim ± SD)^a^			
		Reference method	Proposed methods		
			Method A	Method B	Method C
Suminat-25	25	101.1 ± 0.83	99.52 ± 1.19	101.6 ± 1.45	101.8 ± 1.20
			*t* = 2.44	*t* = 0.67	*t* = 1.07
			*F* = 2.06	*F* = 3.05	*F* = 2.09

Suminat-50	50	99.43 ± 1.05	100.9 ± 0.81	101.2 ± 1.77	99.09 ± 1.83
			*t* = 2.48	*t* = 1.92	*t* = 0.36
			*F* = 0.60	*F* = 2.84	*F* = 3.04

^a^Mean value of five determinations.

Tabulated *t*-value at the 95% confidence level is 2.78.

Tabulated *F*-value at the 95% confidence level is 6.39.

**Table 6:** Results of recovery study by standard addition method.

Tablets studied	Method A				Method B				Method C			
	STS in tablets mg mL^−1^	Pure STS added mg mL^−1^	Total found mg mL^−1^	Pure STS recovered* Percent ± SD	STS in tablets *µ*g mL^−1^	Pure STS added *µ*g mL^−1^	Total found *µ*g mL^−1^	Pure STS recovered* Percent ± SD	STS in tablets *µ*g mL^−1^	Pure STS added *µ*g mL^−1^	Total found *µ*g mL^−1^	Pure STS recovered* Percent ± SD
Suminat-25	2.98	1.5	4.47	99.33 ± 0.07	4.57	2.25	6.87	102.2 ± 1.15	1.02	0.5	1.51	98.20 ± 0.91
	2.98	3.0	6.02	101.3 ± 0.05	4.57	4.50	9.15	101.8 ± 0.93	1.02	1.0	2.04	101.9 ± 1.30
	2.98	4.5	7.60	102.7 ± 0.05	4.57	6.75	11.4	101.2 ± 0.82	1.02	1.5	2.54	101.3 ± 0.89

Suminat-50	3.03	1.5	4.56	102.0 ± 0.05	4.55	2.25	6.78	99.11 ± 1.16	0.99	0.5	1.50	102.4 ± 1.56
	3.03	3.0	6.00	99.00 ± 0.06	4.55	4.50	9.15	102.2 ± 0.68	0.99	1.0	2.00	101.7 ± 0.96
	3.03	4.5	7.60	101.6 ± 0.05	4.55	6.75	11.5	103.0 ± 1.21	0.99	1.5	2.52	102.0 ± 1.03

*Mean value of three determinations.
